# Constructing Well-Being in Organizations: First Empirical Results on Job Crafting, Personality Traits, and Insight

**DOI:** 10.3390/ijerph18126661

**Published:** 2021-06-21

**Authors:** Alessio Gori, Alessandro Arcioni, Eleonora Topino, Letizia Palazzeschi, Annamaria Di Fabio

**Affiliations:** 1 Department of Health Sciences, University of Florence, 50135 Florence, Italy; alessio.gori@unifi.it; 2 Department of Education, Languages, Intercultures, Literatures and Psychology (Psychology Section), University of Florence, 50135 Florence, Italy; alessandro.arcioni25@gmail.com (A.A.); letizia.palazzeschi@unifi.it (L.P.); 3 Department of Human Sciences, LUMSA University of Rome, 00193 Rome, Italy; eleonora.topino@gmail.com

**Keywords:** job crafting, personality traits, insight orientation, mediation analysis, well-being

## Abstract

The construct of job crafting is gaining increasing attention in the research and practice of work psychology in light of the positive effects it has on workers and the organizational context. On this basis, the present study aimed to explore the associations between the Big Five personality traits and job crafting (and its subdimensions, individual job crafting and collaborative job crafting), as well as investigating the role of insight in mediating these relationships. A sample of 159 Italian workers took part in the study and completed the self-report measures. Results showed a positive association between extraversion, agreeableness and conscientiousness and job crafting (total), individual job crafting, and collaborative job crafting, with significant mediations of insight orientation. Openness was positively associated with job crafting (total) and individual job crafting, but not with the collaborative subdimension, with insight orientation that mediated existing relationships. Emotional stability and job crafting (total) or its subdimensions were found to have no significant relationships. These findings suggest that insight orientation could represent a promising resource for job crafting, both in terms of primary prevention, strength-based prevention, and healthy organizations.

## 1. Introduction

The relentless, fast-moving changes of modern times are well known and far from negligible for work environments and organizations [1]. For example, the COVID-19 pandemic has required many workers great adaptability, causing, in some phases, rapid switch in teleworking modalities and distancing from the physical workplace or from face-to-face interaction with colleagues with significant impacts in the world of work [2]. Furthermore, this happened in the context of constant technological advancement, resulting in inevitable obsolescence that forces the labor market to innovate to remain successful and competitive [3,4,5]. Organizations must be flexible and prepared for new, unexpected challenges of our time, and accept that change will come rather than fighting it [6]. As part of adapting, job crafting represents a promising resource for facing these challenges and an interesting, encouraging path to well-being in organizations in the twenty-first century [7,8,9].

Wrzesniewski and Dutton [10] described job crafting as all behaviors that employees can autonomously enact to favor changes in their own working environment and thus reach a better fit between the job and individual preferences, passions, and characteristics. Job crafting has been subsequently defined based on the job demands-resources (J.D.-R.) model [11], in terms of behaviors that employees undertake on their own initiative to make changes to job demands or job resources. In this framework, Tim, Bakker, and Derks [12] referred to four job crafting dimensions: increasing challenging demands, decreasing hindering demands, increasing structural job resources, and increasing social job resources. Recent research identifies functional associations of this construct in the work environment, such as gratitude, social support, job satisfaction [13], and career adaptability (especially for medium and high opportunity-enhancing high-performance work practices) [14].

Although job crafting is often seen as the realm of individuals, it can benefit from collaboration [15,16]. The construct of individual job crafting is enriched by introducing the dimension of collaborative job crafting [16], the set of behaviors that groups of coworkers collaboratively enact to craft their own work as one. Social interactions are the most relevant in this specific job crafting domain; particularly, the frequency of such interactions and closeness among colleagues may predict collaborative job crafting skills [16]. Workers may spontaneously cooperate in crafting their job to better fit job tasks to their own interests and skills. Collaborative job crafting may be explicit or implicit. In fact, workers may spontaneously craft their jobs despite having not previously planned an effective change [16,17]. Furthermore, individual and collaborative job crafting are not mutually exclusive [16,17].

Given the presented evidence concerning its applicability to the working world, the general aim of this research was to provide more in-depth knowledge concerning the antecedents to job crafting and its subdimensions, by focusing on insight orientation and personality traits.

Indeed, the impact and effect of job crafting are influenced by employee perception and their attitudes [10,16,18,19]. In this regard, Rudolph, Katz, Lavigne, and Zacher [18] further explored individual antecedents of job crafting through a meta-analysis. The authors proposed a comprehensive model in which they included the Big Five personality traits as dimensions that play the role of antecedents of job crafting [18]. In particular, extraverted workers, who are friendly and comfortable in social interactions [20], seem to enhance proactive behavior in relational contexts [21]. Workers with high agreeableness scores and who are friendly and cooperative seem to be able to increase social job resources, similarly to extraverted individuals [18]. Conscientious workers, who are task-oriented and persistent, seem to be more inclined to challenge job demands and diminish the impact of workloads [18]. Employees with low neuroticism scores tend to feel positive emotions. They seem to cope better with stressors than workers with high scores, resulting in a weaker impact on their psychological well-being [22] and a sense of self-efficacy in promoting change [23]. Finally, employees who are open to experience, curious, and creative may enact proactive behaviors, especially during the information collection phase [24]. As the scientific literature has shown that personality traits are stable over time, it would be interesting to conduct further research to understand better which variables mediate the association between personality traits and job crafting.

In this theoretical framework, insight could be one such promising variable. Insight is a complex construct defined as “insight is a dynamic variable that ranges from superficial consciousness to complex comprehension of elaborated emotional material” [25] (p. 299). It was born as an aspect linked to the psychotherapeutic field but has also shown strong promise in work psychology research. For example, good insight was associated with workplace relational civility [26], which represents a larger construct that includes emotional intelligence [27], and is the cornerstone of the awareness of the need for change: it involves reflective functioning skills, which provide emotional awareness, problem-solving skills, and the predisposition to change [25,26,28,29]. Although it has traditionally been difficult to alter employees’ personality traits, since traits tend to be stable over a lifetime [30], it might be possible to increase insight orientation through improvements in relational civility [27], experience in various spheres of life, and, sometimes, through specific training. According to the strength-based prevention perspective [31], improving employees’ individual resources may enhance workers’ strengths to help them cope with job demands and improve feelings of well-being at work. Thus, insight orientation emerges as a promising variable of study not only in the psychotherapeutic field but also in work and organizational psychology to explore the relationships between personality and job crafting.

Given these assumptions, the present study aims to explore the relationships between personality traits, job crafting, and insight orientation through several mediation models. In particular, we assume that personality traits can be related to job crafting, that personality traits can influence insight orientation, and that insight orientation can be associated with job crafting. We tested several mediation models based on these assumptions, using the five personality traits as independent variables, job crafting and its subcomponents as dependent variables, and insight orientation as the mediating variable.

## 2. Methods

### 2.1. Participants and Procedure

A group of 159 participants (82 males and 77 females; mean age 50.88 years; SD = 8.99) took part in the study. All of them were employees of organizations in Tuscany, Italy, and voluntarily participated in the study, without obtaining any form of compensation. Before starting, all participants were informed about the general purpose of the study and gave written informed consent that specified their right to quit at any time. The self-report measures were administered by trained psychologists, which counterbalanced the sequence of the questionnaires to control for the potential effect of presentation. The research was conducted under Italian privacy, informed consent laws, and the procedure met ethical standards approved by the Ethics Committee of the Integrated Psychodynamic Psychotherapy Institute (IPPI).

### 2.2. Measures

#### 2.2.1. Job Crafting Scale (JCS)

The Italian version [17] of the Job Crafting Scale [16] was used. It is composed of 12 items on a scale from 1 = never to 5 = always. The scale included a total score and an assessment of two subdimensions, each evaluated with six mirror items that differ only in the presence of “on my own” and “together.” For example, one question under individual job crafting says, “On your own, change the way you do your job to make it easier to yourself,” while the same question under collaborative job crafting says, “Decide together with your coworkers to change the way you do your job to make it easier to yourself.” Adding up the subdimension scores gives us the scores for each dimension of the JCS, and adding those gives us the total score. The Italian version of the JCS showed good reliability (α = 0.92 for the total score, α = 0.85 for individual job crafting, and α = 0.93 for collaborative job crafting).

#### 2.2.2. Big Five Questionnaire (BFQ)

The Big Five Questionnaire [32] includes 132 items on a scale from 1 = absolutely false to 5 = absolutely true. The scale focuses on the five principal personality dimensions and their 10 subdimensions: (1) extraversion, which includes the factors of dynamism and dominance; (2) agreeableness, which includes the factors of cooperativeness and cordiality; (3) conscientiousness, which includes the factors of scrupulousness and perseverance; (4) emotional stability, which includes the factors of emotional control and impulse control; and (5) openness, which includes the factors of cultural openness and openness to experience. The Italian version of the instrument had good psychometric properties and internal consistency reliability [32], showing satisfactory Cronbach’s alpha coefficients (with α ranging from 0.73 for agreeableness to 0.90 for emotional stability).

#### 2.2.3. Insight Orientation Scale (IOS)

The Insight Orientation Scale (IOS) [25] is a 7-item measure assessing clients’ capacity for insight and also comprising their behaviors, feelings, and opinions about it. The response format ranges from 1 = not at all to 5 = a great deal (e.g., “I am able to be reflective about myself”). Although the items cover different theoretical dimensions of insight orientation (awareness, problem solving, surprise, complexity, level of consciousness, restructuring, self-reflectiveness), the IOS has no subscales and showed good reliability (α = 0.77) [25].

### 2.3. Statistical Analyses

All statistical analyses were performed using SPSS for Windows (version 26.0, IBM, Armonk, NY, USA). Descriptive statistics for both the samples and variables were computed. Pearson’s r correlations were performed to assess the relationships between the dimensions of interest in the study. The mediation models were tested using the SPSS macro program PROCESS v3.5 [33]. Several single mediations (Model 4) were carried out to verify the mediating role of insight orientation in the association between each personality trait and job crafting. Then, the same procedure was performed to assess the mediating role of insight orientation in the causal relationship between each personality trait and both subcomponents of job crafting (individual and collaborative). The indirect effect was tested employing bootstrapping procedures with a 95% confidence interval for 5000 samples.

## 3. Results

Descriptive statistics and correlations between the BFQ, IOS, and JCS are presented in Table 1.

Regarding the mediation analyses with extraversion as the independent variable, the data showed that insight orientation mediated the relationship between extraversion and the total job crafting score, as well as between extraversion and both individual and collaborative job crafting.

**Figure 1:**
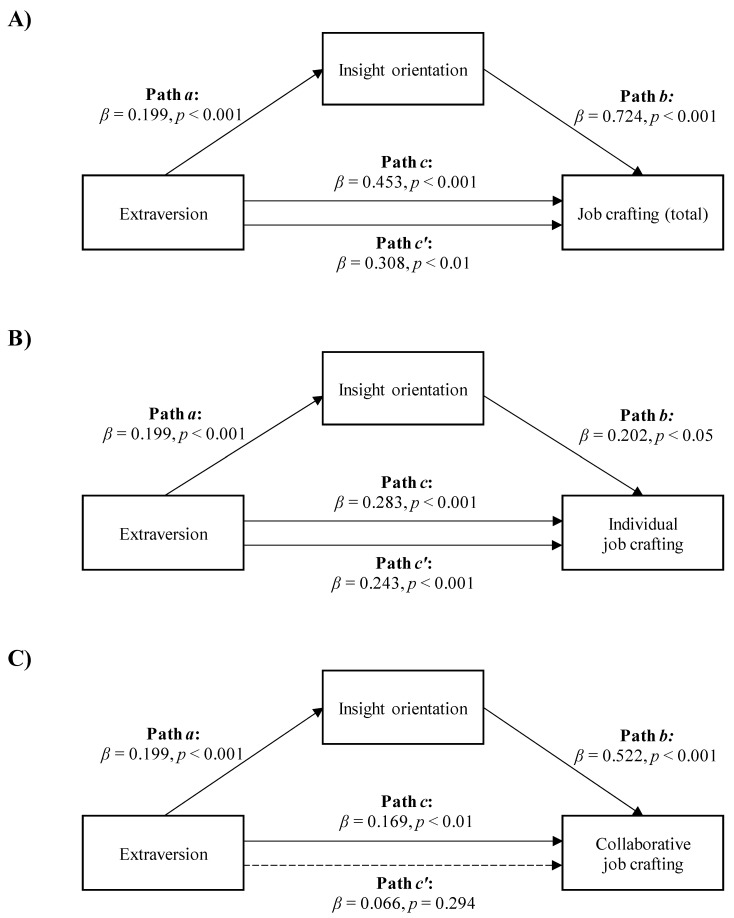
The mediation of insight orientation in the relationships between extraversion and total job crafting score (**A**) *R*^2^ mediator effect size = 0.12, *p* < 0.01), individual job crafting, (**B**) *R*^2^ mediator effect size = 0.06, *p* < 0.05) or collaborative job crafting (**C**) *R*^2^ mediator effect size = 0.14, *p* < 0.01).

Extraversion was positively and directly related to the total job crafting score and indirectly related to the total job crafting score through insight orientation. Figure 1A shows that workers with higher extraversion scored higher on insight orientation (Path *a*; *β* = 0.199, *p* < 0.001), which, in turn, was related to a higher total job crafting score (Path *b*; *β* = 0.724, *p* < 0.001). A bias-corrected bootstrap confidence interval for the indirect effect (Path a*b; *β* = 0.144) based on 5000 bootstrap samples was entirely above zero (0.063–0.235). The association between extraversion and total job crafting score was reduced after controlling for insight orientation but remained statistically significant (Path *c′* in Figure 1A; *β* = 0.308, *p* < 0.01). This showed a partial mediation model with *R*^2^ mediator effect size = 0.12 and *p* < 0.01.

The second mediation model suggested a positive association between extraversion and individual job crafting, both directly and indirectly, through insight orientation. Figure 1B shows that higher extraversion in workers was associated with higher insight orientation (Path *a*; *β* = 0.199, *p* < 0.001), which, in turn, was related to higher individual job crafting scores (Path *b*; *β* = 0.202, *p* < 0.05). A bias-corrected bootstrap confidence interval for the indirect effect (Path a*b; *β* = 0.040) based on 5000 bootstrap samples was above zero (0.001–0.084). The effect of extraversion on individual job crafting was reduced after controlling for insight orientation, but remained significant (Path c′ in Figure 1B; *β* = 0.243, *p* < 0.001). This indicated a partial mediation model with *R*^2^ mediator effect size = 0.06 and *p* < 0.05.

Third, extraversion was positively related to collaborative job crafting, both directly and indirectly, through insight orientation. Figure 1C shows that higher extraversion in workers was associated with higher insight orientation (Path *a*; *β* = 0.199, *p* < 0.001), which, in turn, was related to higher collaborative job crafting scores (Path *b*; *β* = 0.522, *p* < 0.001). A bias-corrected bootstrap confidence interval for the indirect effect (Path a*b; *β* = 0.104) based on 5000 bootstrap samples was entirely above zero (0.052–0.166). The effect of extraversion on collaborative job crafting was reduced after controlling for insight orientation and became insignificant (Path *c′* in Figure 1C; *β* = 0.066, *p* = 0.294). This showed a total mediation model with *R*^2^ mediator effect size = 0.14 and *p* < 0.01.

Regarding the mediation analyses with agreeableness as the independent variable, the data showed that insight orientation mediated the relationship between agreeableness and the total job crafting score, as well as between agreeableness and both individual and collaborative job crafting.

**Figure 2:**
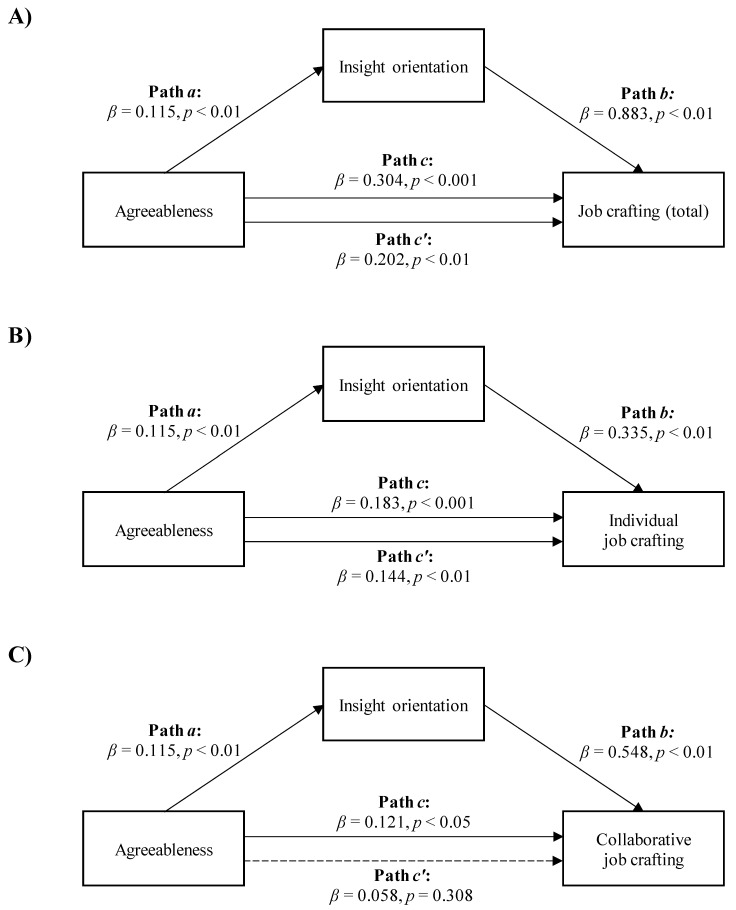
The mediation of insight orientation in the relationships between agreeableness and total job crafting score. (**A**) *R*^2^ mediator effect size = 0.09, (*p* < 0.05), individual job crafting (**B**) *R*^2^ mediator effect size = 0.06, (*p* < 0.05) or collaborative job crafting, (**C**) *R*^2^ mediator effect size = 0.09, (*p* < 0.05).

Agreeableness was positively and directly related to the total job crafting score and indirectly related to the total job crafting score through insight orientation. As shown in Figure 2A, workers with higher agreeableness scored higher on insight orientation (Path *a*; *β* = 0.115, *p* < 0.01), which, in turn, was related to a higher total job crafting score (Path *b*; *β* = 0.883, *p* < 0.01). A bias-corrected bootstrap confidence interval for the indirect effect (Path a*b; *β* = 0.102) based on 5000 bootstrap samples was entirely above zero (0.035–0.178). The effect of agreeableness on the total job crafting score was reduced after controlling for insight orientation, but remained statistically significant (Path *c′* in Figure 2A; *β* = 0.202, *p* < 0.05). This indicated a partial mediation model with *R*^2^ mediator effect size = 0.09 and *p* < 0.05.

The second mediation suggested a positive association between agreeableness and individual job crafting, both directly and indirectly, through insight orientation. Figure 2B shows that higher agreeableness in workers was associated with higher insight orientation (Path *a*; *β* = 0.115, *p* < 0.01), which, in turn, was related to higher individual job crafting scores (Path *b*; *β* = 0.335, *p* < 0.01). A bias-corrected bootstrap confidence interval for the indirect effect (Path a*b; *β* = 0.039) based on 5000 bootstrap samples was above zero (0.012–0.073). The effect of agreeableness on individual job crafting was reduced after controlling for insight orientation, although it remained significant (Path *c′* in Figure 2B; *β* = 0.144, *p* < 0.01). This indicated a partial mediation model with *R*^2^ mediator effect size = 0.06 and *p* < 0.05.

Third, agreeableness was positively related to collaborative job crafting, both directly and indirectly, through insight orientation. Figure 2C shows that higher agreeableness in workers was associated with higher insight orientation (Path *a*; *β* = 0.115, *p* < 0.01), which, in turn, was related to higher collaborative job crafting scores (Path *b*; *β* = 0.548, *p* < 0.01). A bias-corrected bootstrap confidence interval for the indirect effect (ab = 0.063) based on 5000 bootstrap samples was entirely above zero (0.022–0.112). The effect of agreeableness on collaborative job crafting was reduced after controlling for insight orientation and became insignificant (Path *c′* in Figure 2C; *β* = 0.058, *p* = 0.308). This indicated a total mediation model with *R*^2^ mediator effect size = 0.09 and *p* < 0.05.

Regarding the mediation analyses with conscientiousness as the independent variable, the data showed that insight orientation mediated the relationship between conscientiousness and the total job crafting score, as well as between conscientiousness and both individual and collaborative job crafting.

**Figure 3:**
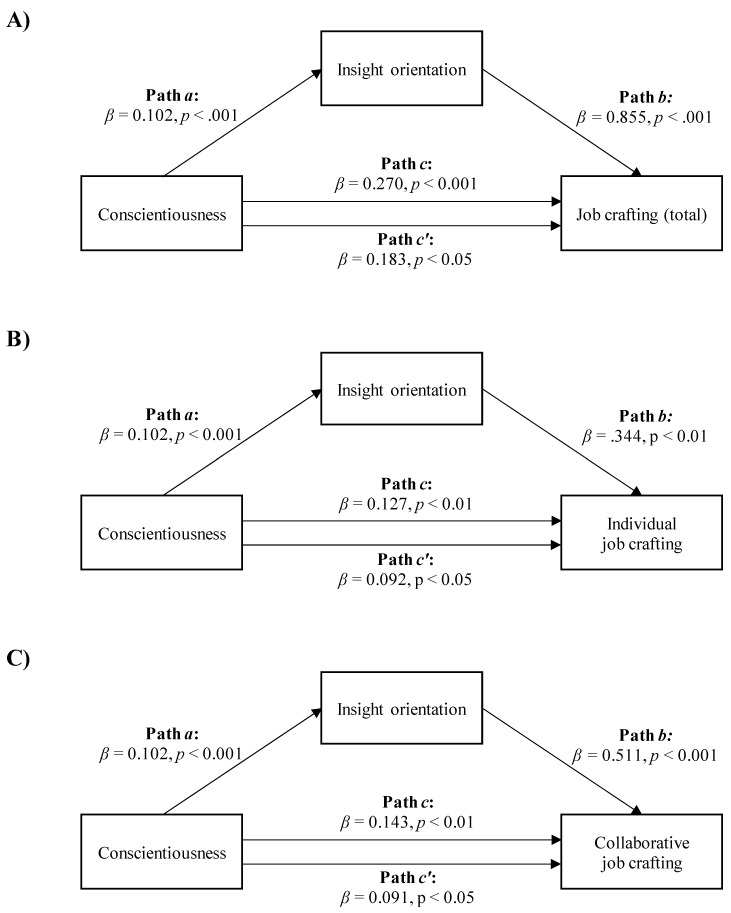
The mediation of insight orientation in the relationships between conscientiousness and total job crafting score. (**A**) *R*^2^ mediator effect size = 0.09, (*p* < 0.05), individual job crafting (**B**) *R*^2^ mediator effect size = 0.07, (*p* < 0.05) or collaborative job crafting; (**C**) *R*^2^ mediator effect size = 0.09, (*p* < 0.05).

Conscientiousness was positively and directly related to the total job crafting score and indirectly to the total job crafting score through insight orientation. As shown in Figure 3A, workers with higher conscientiousness scored higher on insight orientation (Path *a*; *β* = 0.102, *p* < 0.001), which, in turn, was related to higher total job crafting scores (Path *b*; *β* = 0.855, *p* < 0.001). A bias-corrected bootstrap confidence interval for the indirect effect (Path a*b; *β* = 0.087) based on 5000 bootstrap samples was entirely above zero (0.033–0.168). The effect of conscientiousness on the total job crafting score was reduced after controlling for insight orientation, but remained statistically significant (Path *c′* in Figure 3A; *β* = 0.183, *p* < 0.05). This indicated a partial mediation model with *R*^2^ mediator effect size = 0.09 and *p* < 0.05.

The second mediation model suggested a positive relationship between conscientiousness and individual job crafting, both directly and through insight orientation. Figure 3B shows that higher conscientiousness in workers was associated with higher insight orientation (Path *a*; *β* = 0.102, *p* < 0.001), which, in turn, was related to higher individual job crafting scores (Path *b*; *β* = 0.344, *p* < 0.01). A bias-corrected bootstrap confidence interval for the indirect effect (Path a*b; *β* = 0.350) based on 5000 bootstrap samples was above zero (0.010–0.074). The effect of conscientiousness on individual job crafting was reduced after controlling for insight orientation, but remained significant (Path *c′* in Figure 3B; *β* = 0.092, *p* < 0.05). This indicated a partial mediation model with *R*^2^ mediator effect size = 0.07 and *p* < 0.05.

Third, conscientiousness was positively related to collaborative job crafting, both directly and indirectly through insight orientation. As shown in Figure 3C, higher conscientiousness in workers was associated with higher insight orientation (Path *a*; *β* = 0.102, *p* < 0.001), which, in turn, was associated with higher collaborative job crafting scores (Path *b*; *β* = 0.511, *p* < 0.001). A bias-corrected bootstrap confidence interval for the indirect effect (Path a*b; *β* = 0.052) based on 5000 bootstrap samples was entirely above zero (0.020–0.099). The effect of conscientiousness on individual job crafting was reduced after controlling for insight orientation, but remained significant (Path *c′* in Figure 3C; *β* = 0.091, *p* < 0.05). This indicated a partial mediation model with *R*^2^ mediator effect size = 0.09 and *p* < 0.05.

Regarding the mediation analyses with emotional stability as the independent variable, the data showed that insight orientation did not contribute to mediating the association between emotional stability and the total job crafting score or between emotional stability and either individual or collaborative job crafting.

**Figure 4:**
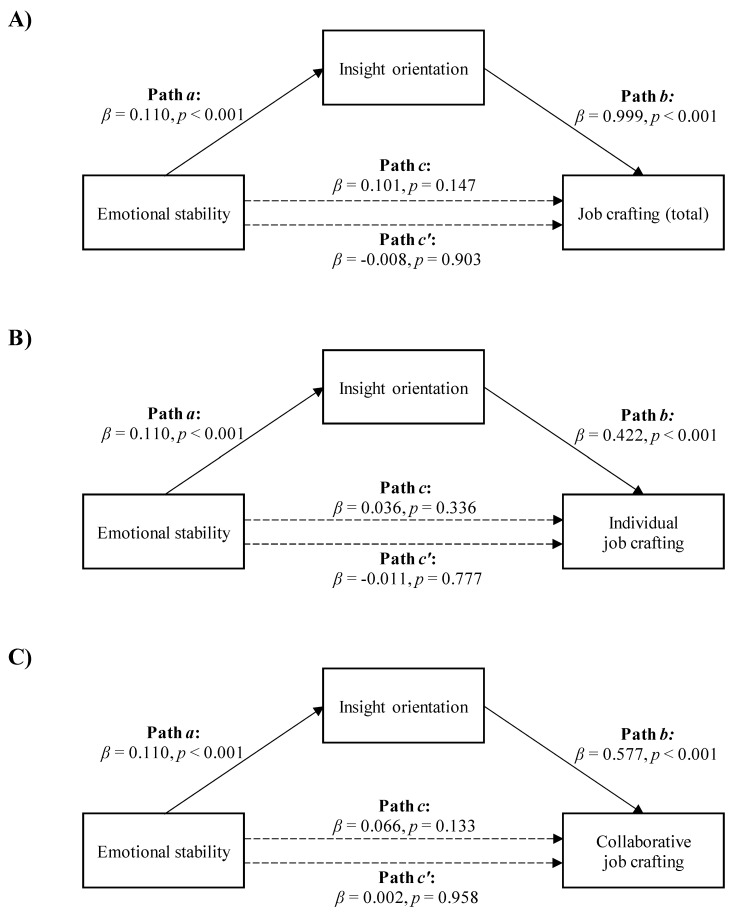
The mediation of insight orientation in the relationships between emotional stability and total job crafting score: (**A**) no mediation, individual job crafting; (**B**) no mediation or collaborative job crafting; (**C**) no mediation.

First, insight orientation did not contribute to mediating the association between emotional stability and the total job crafting score (Path *c* in Figure 4A, *β* = 0.101, *p* = 0.147; Path *c’*, *β* = -0.008, *p* = 0.903).

Second, insight orientation did not contribute to mediating the association between emotional stability and individual job crafting (Path *c* in Figure 4B, *β* = 0.036, *p* = 0.336; Path *c’*, *β* = -0.011, *p* = 0.777).

Third, insight orientation did not contribute to mediating the association between emotional stability and collaborative job crafting (Path *c* in Figure 4C, *β* = 0.066, *p* = 0.133; Path *c’*, *β* = 0.002, *p* = 0.958).

Finally, regarding mediation analyses with openness as the independent variable, the data showed that insight orientation mediated the relationship between openness and the total job crafting score, as well as between openness and individual job crafting, but it did not contribute to mediating the association between openness and collaborative job crafting.

**Figure 5:**
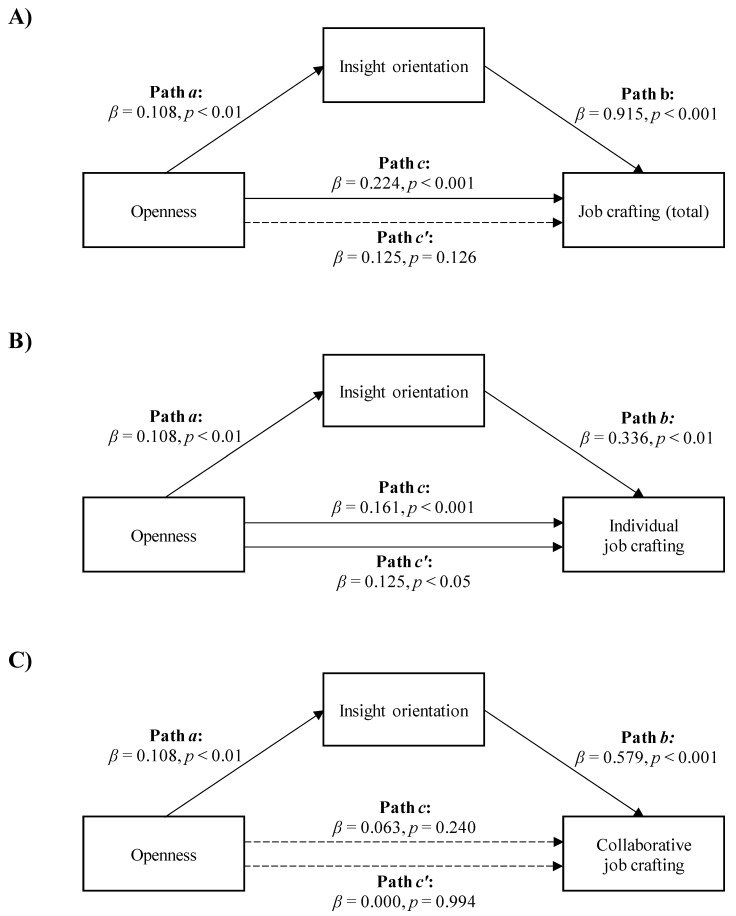
The mediation of insight orientation in the relationships between openness and total job crafting score. (**A**) *R*^2^ mediator effect size = 0.09, (*p* < 0.05), individual job crafting (**B**) *R*^2^ mediator effect size = 0.06, (*p* < 0.05) or collaborative job crafting; (**C**) no mediation.

Openness was positively and directly related to the total job crafting score and indirectly to the total job crafting score through insight orientation. As shown in Figure 5A, workers with higher openness scored higher on insight orientation (Path *a*; *β* = 0.108, *p* < 0.01), which, in turn, was related to higher total job crafting scores (Path *b*; *β* = 0.915, *p* < 0.001). A bias-corrected bootstrap confidence interval for the indirect effect (Path a*b; *β* = 0.099) based on 5000 bootstrap samples was entirely above zero (0.035–0.185). The effect of openness on the total job crafting score was reduced after controlling for insight orientation and became statistically insignificant (Path c′ in Figure 5A; *β* = 0.125, *p* = 0.126). This indicated a total mediation model with *R*^2^ mediator effect size = 0.09 and *p* < 0.01.

The second mediation model suggested a positive association between openness and individual job crafting, both directly and indirectly, through insight orientation. Figure 5B shows that higher openness in workers was associated with higher insight orientation (Path *a*; *β* = 0.108, *p* < 0.01), which, in turn, was associated with higher scores in individual job crafting (Path *b*; *β* = 0.336, *p* < 0.01). A bias-corrected bootstrap confidence interval for the indirect effect (Path a*b; *β* = 0.036) based on 5000 bootstrap samples was above zero (0.11–0.72). The effect of openness on individual job crafting was reduced after controlling for insight orientation, but remained significant (Path *c′* in Figure 5B; *β* = 0.125, *p* < 0.05). This indicated a partial mediation model with *R*^2^ mediator effect size = 0.06 and *p* < 0.05.

Third, insight orientation did not contribute to mediating the association between openness and collaborative job crafting (Path *c* in Figure 5C, *β* = 0.063, *p* = 0.240; Path *c’*, *β* = 0.000, *p* = 0.995).

## 4. Discussion

In recent times, many advancements and changes have influenced work environments due to an ever-more-high-tech world with fewer boundaries and more globalized market systems [1]. New technologies have also caused work to intermingle more frequently with personal life [34,35,36], representing a challenge for competitive organizations [37]. Such uncertainty and loss of boundaries require empowered employees to face feelings of discouragement, bewilderment, and stress [38]. Hence, it is important to detect personal resources that promote both individual and organizational well-being and productivity.

In this study, we also attempted to emphasize the primary prevention [39,40,41,42] and strength-based prevention perspectives [31]. In line with this framework, this study examined the role of insight orientation as a promising variable in mediating the relationship between personality traits and job crafting among Italian workers. We conducted several mediation models to verify the contribution of insight orientation to the association between personality traits and individual and collaborative job crafting. Our results showed that insight orientation could be a key mediator in the associations between some personality traits and job crafting. Although personality traits contributed directly to job crafting, there was also a pathway via insight orientation; in particular, individuals with extraversion, agreeableness, conscientiousness, and openness were not only more able to craft their job but also have greater insight skills, which, in turn, increased their job crafting abilities.

It is conceivable that extraverted people may be sociable and enjoy stimulating activities [43], and therefore, this tendency to prove themselves could lead to reaching new awareness; those agreeable people may be inclined to self-reflection thanks to their tendency to be attuned to others [44]; that conscientious people are scrupulous and responsible [45] and so perhaps have a deeper interest in reflecting better fit with incoming demands; and that open-minded people have greater insight orientation precisely because of their tendency toward intellectualism, independence of mind, and reflection [46].

Therefore, our findings highlighted the contribution of insight orientation to mediating the relationship between these personality traits and job crafting. Insight includes flexibility and open-minded attitudes that could positively contribute to job crafting [25,28,29].

More specifically, insight orientation mainly mediated the association between extraversion and job crafting, suggesting that this construct could contribute assertiveness and energy toward the ability to craft one’s job according to one’s preferences, passions, and needs. The association of extraversion with job crafting was consistent with the conceptualization of extraversion itself, a personality trait linked to high levels of energy, sociability, enthusiasm, initiative, and assertiveness [18,43]. Therefore, it is appropriate to deduce that extraverted workers have the skills to craft their own jobs. Particularly, the mediating role of insight orientation seems to be stronger in the relationship between extraversion and the collaborative dimension, suggesting that awareness and inclination to reflection are helpful for extraverted workers engaging in collaborative job crafting.

Moreover, insight orientation also mediated the relationships of agreeableness, conscientiousness, and openness with job crafting but not with emotional stability. In particular, insight orientation mediated the relationship between agreeableness and job crafting (total and both individual and collaborative). Individuals who are more cooperative and cordial tend more toward job crafting. Agreeableness was thus positively related to job crafting, also indirectly through the positive effect of insight orientation. This was particularly true for collaborative job crafting (total mediation) since more agreeable people could self-reflect more with to harmony with others and seem more inclined to collaborate with others to craft their jobs.

Insight orientation also partially mediated the relationship between conscientiousness and job crafting (total, both individual and collaborative), revealing that more scrupulous and perseverant individuals seem to show more job crafting. Still, there was also an indirect relationship through insight orientation. Participants with a higher level of conscientiousness are more accurate, precise, task-oriented, and persistent and thus likely have a greater tendency to reflect on themselves and on the processes, they are involved in. They also seemed to be more inclined to craft their jobs when faced with challenges.

In our study, emotional stability was shown not to be associated with job crafting, a result that is discordant with other studies in the literature [18]. In all likelihood, emotionally stable workers are not as engaged in changing their status quo and thus perhaps tend not to craft their job. On the other hand, workers with high levels of extraversion, agreeableness, conscientiousness, and openness tend to craft their job due to their corresponding tendency to bend to others’ mental states, be careful about their performance, and seek novelty [18].

Another interesting result was that insight orientation completely mediated the relationship between openness and total job crafting score and partially between openness and individual job crafting, while openness did not mediate collaborative job crafting. Curious, intellectual, and independent-minded workers did not seem inclined to look for collaborative ways to craft their job, preferring individual approaches. Conversely, the fact that the direct effect of openness became statistically insignificant highlights the essential contribution of insight orientation to promoting job crafting.

This research has numerous limitations that should be taken into consideration. First, it involved a non-representative sample of Italian workers, which might influence the ability to generalize our findings. Further research may want to consider a larger sample by recruiting workers from other geographical areas in Italy. Furthermore, no distinctions were made between job types, which could be an important issue that could influence results. Therefore, an important challenge for future research could be the exploration of the relationship between personality traits, insight orientation and job crafting in workers also differentiating for job types. Finally, the study could not definitively establish causal links among variables, since it was not longitudinal. Future research could, thus, also examine these relationships through a longitudinal study.

## 5. Conclusions

This study reveals some important reflections about certain variables involved in increasing organizational and worker productivity and wellness. In fact, it highlights the contribution of insight orientation in mediating the association between personality traits and job crafting, thus broadening the acknowledgment of the association between dispositional and job characteristics, which in turn influences organizational productivity and outcomes, as well as job-person fit [47].

These results can be of great value in a “healthy organization” perspective [48,49,50], which supports virtuous organizational culture and climate [51], tends to encourage proactive behaviors and the balance the relationship between job demands and one’s own motivation and expectations [47,52], with important implications for employee and organization well-being. More specifically, since it is implicated in mediating associations between personality traits and job crafting, insight orientation could represent a critical individual resource amenable to training, contrary to personality traits that have been deemed as substantially stable in the literature [30]. However, previous scientific literature showed that some personality traits could be more fertile ground than others in promoting a state of improvement. For example, by increasing the use of coping strategies appropriate to the situation [53] and being linked to the use of defense mechanisms that can be more or less functional [54], both fundamental aspects for stress management [55]. On that basis, the improvement of insight orientation can allow the acquisition of new awareness (of cognitive, emotional and social aspects) that can put jobs in a condition that facilitates job crafting and that are also linked to benefit on well-being and health: awareness, both self-oriented and towards others, is in fact associated with better functioning [56]. Furthermore, scientific literature showed that both job crafting and personality traits had significant relationships with leadership and strategic human resource management (HRM) [19,57]. Therefore, the present study results may be useful for the implementation of practice intervention on job crafting, integrating them with previous evidence identifying transformational leadership styles [58,59] or responsible leadership [60,61] as prominent elements of this perspective.

Summing up, if the findings of this research will be confirmed in future studies, the value of early intervention in implementing insight orientation in relation to job crafting could be considered. This could lead researchers to consider the critical importance of improving insight orientation in workers through specific training in organizations starting at the initial stages of organizational socialization based on the primary prevention [39,40,41,42] and strength-based prevention perspectives [31]. This study highlights the importance of studying insight orientation in-depth as a highly promising resource for facilitating job crafting in the challenging and unpredictable twenty-first century world of work, one more opportunity to promote well-being in organizations [48,49,62].

## Figures and Tables

**Table 1:** Correlations among BFQ dimensions, IOS and JCS (*N* = 159).

	1	2	3	4	5	6	7	8	9
1. BFQ Extraversion	1								
2. BFQ Agreeableness	0.32 **	1							
3. BFQ Conscientiousness	0.24 **	0.22 **	1						
4. BFQ Emotional stability	0.29 **	0.33 **	0.03	1					
5. BFQ Openness	0.38 **	0.39**	0.46 **	0.07	1				
6. IOS Insight Orientation	0.41 **	0.25 **	0.28 **	0.32 **	0.26 **	1			
7. JCS Job Crafting	0.37 **	0.26 **	0.29 **	0.12	0.21 **	0.39 **	1		
8. JCS Individual Crafting	0.43 **	0.29 **	0.25 **	0.08	0.28 **	0.31 **	0.84 **	1	
9. JCS Collaborative Crafting	0.22 **	0.16 *	0.24 **	0.12	0.09	0.36 **	0.89 **	0.50 **	1
M	76.01	79.08	83.14	72.52	81.33	25.96	43.61	21.08	22.53
SD	9.27	9.58	12.04	12.94	10.57	4.46	11.36	6.01	7.10

* Correlation is significant at the 0.05 level (two-tailed); ** correlation is significant at the 0.01 level (two-tailed); BFQ = Big Five Questionnaire; IOS = Insight Orientation Scale; JCS = Job Crafting Scale; M = mean; SD = standard deviation.

## Data Availability

The data presented in this study are available on request from the corresponding author. The data are not publicly available due to privacy reasons.

## References

[1] Blustein D.L., Kenny M.E., Di Fabio A., Guichard J. (2019). Expanding the impact of the psychology of working: Engaging psychology in the struggle for decent work and human rights. J. Career Assess..

[2] Belzunegui-Eraso A., Erro-Garcés A. (2020). Teleworking in the Context of the Covid-19 Crisis. Sustainability.

[3] Areed S., Salloum S.A., Shaalan K., Al-Emran M., Shaalan K., Hassanien A.E. (2020). The role of knowledge management processes for enhancing and supporting innovative organizations: A systematic review. Recent Advances in Intelligent Systems and Smart Applications.

[4] Khosravi P., Newton C., Rezvani A. (2019). Management innovation: A systematic review and meta-analysis of past decades of research. Eur. Manag. J..

[5] Manuti A., De Palma P.D. (2016). The Social Organization: Managing Human Capital Through Social Media.

[6] Di Fabio A., Gori A. (2016). Developing a new instrument for assessing acceptance of change. Front. Psychol..

[7] Boehnlein P., Baum M. (2020). Does job crafting always lead to employee well-being and performance? Meta-analytical evidence on the moderating role of societal culture. Int. J. Hum. Res. Manag..

[8] Tims M., Bakker A.B., Derks D. (2013). The impact of job crafting on job demands, job resources, and well-being. J. Occup. Health Psych..

[9] Van Wingerden J., Bakker A.B., Derks D. (2017). Fostering employee well-being via a job crafting intervention. J. Voc. Behav..

[10] Wrzesniewski A., Dutton J.E. (2001). Crafting a job: Revisioning employees as active crafters of their work. Acad. Manag. Rev..

[11] Demerouti E., Bakker A.B., Nachreiner F., Schaufeli W.B. (2001). The job demands-resources model of burnout. J. Appl. Psych..

[12] Tims M., Bakker A.B., Derks D. (2012). Development and validation of the job crafting scale. J. Voc. Behav..

[13] Chen H., Yang X., Xia W., Li Y., Deng Y., Fan C. (2021). The relationship between gratitude and job satisfaction: The mediating roles of social support and job crafting. Curr. Psychol..

[14] Federici E., Boon C., Den Hartog D.N. (2021). The moderating role of HR practices on the career adaptability–job crafting relationship: A study among employee–manager dyads. Int. J. Hum. Resour. Manag..

[15] Alonso C., Fernández-Salinero S., Topa G. (2019). The impact of both individual and collaborative job crafting on Spanish teachers’ well-being. Edu. Sci..

[16] Leana C., Appelbaum E., Shevchuk I. (2009). Work process and quality of care in early childhood education: The role of job crafting. Acad. Manag. J..

[17] Di Fabio A. (2020). Job Crafting Scale: Proprietà psicometriche della versione italiana. Counssling.

[18] Rudolph C.W., Katz I.M., Lavigne K.N., Zacher H. (2017). Job crafting: A meta-analysis of relationships with individual differences, job characteristics, and work outcomes. J. Voc. Behav..

[19] Jiang F., Lu S., Wang H., Zhu X., Lin W. (2021). The Roles of Leader Empowering Behaviour and Employee Proactivity in Daily Job Crafting: A Compensatory Model. Eur. J. Work Organ. Psychol..

[20] Asendorpf J.B., Wilpers S. (1998). Personality effects on social relationships. J. Pers. Soc. Psych..

[21] Wu C.H., Li W.D., Parker S.K., Bindl U.K. (2017). Individual differences in proactivity: A developmental perspective. Proactivity at Work: Making Things Happen in Organizations.

[22] Judge T.A., Locke E.A., Durham C.C., Kluger A.N. (1998). Dispositional effects on job and life satisfaction: The role of core evaluations. J. Appl. Psych..

[23] Morrison E.W., Phelps C.C. (1999). Taking charge at work: Extrarole efforts to initiate workplace change. Acad. Manag. J..

[24] Frese M., Fay D. (2001). Personal initiative: An active performance concept for work in the 21st century. Res. Org. Behav..

[25] Gori A., Craparo G., Giannini M., Loscalzo Y., Caretti V., La Barbera D., Schuldberg D. (2015). Development of a new measure for assessing insight: Psychometric properties of the insight orientation scale (IOS). Schizoph. Res..

[26] Gori A., Topino E. (2020). Predisposition to Change Is Linked to Job Satisfaction: Assessing the Mediation Roles of Workplace Relation Civility and Insight. Int. J. Environ. Res. Public Health.

[27] Di Fabio A., Gori A. (2016). Decent work and positive relational outcomes: Assessing Workplace Relational Civility (WRC) with a new multidimensional “mirror” measure. Front. Psychol..

[28] Hays R.B., Jolly B.C., Caldon L.J.M., McCrorie P., McAvoy P.A., McManus I.C., Rethans J.J. (2002). Is insight important? Measuring capacity to change performance. Med. Educ..

[29] Rangell L. (1981). From insight to change. J. Am. Psychol. Ass..

[30] Costa P.T., McCrae R.R. (1992). NEO PI-R Professional Manual.

[31] Di Fabio A., Saklofske D.H. (2021). The relationship of compassion and self-compassion with personality and emotional intelligence in organizations. Personal. Individ. Differ..

[32] Caprara G.V., Barbaranelli C., Borgogni L., Perugini M. (1993). The “Big Five Questionnaire”: A new questionnaire to assess the five factor model. Personal. Individ. Differ..

[33] Hayes A.F. (2018). Introduction to Mediation, Moderation, and Conditional Process Analysis Second Edition: A Regression-Based Approach.

[34] Duffy R.D., Kim H.J., Gensmer N.P., Raque-Bogdan T.L., Douglass R.P., England J.W., Buyukgoze-Kavas A. (2019). Linking decent work with physical and mental health: A psychology of working perspective. J. Voc. Behav..

[35] MacDermid S.M., Kossek E.E., Lambert S.J. (2004). Considering conflict between work and family. Work and Life Integration: Organizational, Cultural, and Individual Perspectives.

[36] Sayah S. (2013). Managing work–life boundaries with information and communication technologies: The case of independent contractors. New Tech. Work Employ..

[37] Murray A., Rhymer J., Sirmon D.G. (2020). Humans and technology: Forms of conjoined agency in organizations. Acad. Manag. Rev..

[38] Scherer S. (2009). The Social Consequences of Insecure Jobs. Soc. Indic. Res..

[39] Di Fabio A., Kenny M.E. (2016). From decent work to decent lives: Positive Self and Relational Management (PS&RM) in the twenty-first century. Front. Psychol..

[40] Di Fabio A., Kenny M.E. (2018). Connectedness to nature, personality traits and empathy from a sustainability perspective. Curr. Psychol..

[41] Hage S.M., Romano J.L., Conyne R.K., Kenny M., Matthews C., Schwartz J.P., Waldo M. (2007). Best practice guidelines on prevention practice, research, training, and social advocacy for psychologists. Couns. Psychol..

[42] Kenny M.E., Hage S.M. (2009). The next frontier: Prevention as an instrument of social justice. J. Prim. Prev..

[43] Lucas R.E., Diener E., Grob A., Suh E.M., Shao L. (2000). Cross-cultural evidence for the fundamental features of extraversion. J. Pers. Soc. Psychol..

[44] Wilmot M.P., Wanberg C.R., Kammeyer-Muelle J.D., Ones D.S. (2019). Extraversion advantages at work: A quantitative review and synthesis of the meta-analytic evidence. J. Appl. Soc. Psychol..

[45] Roberts B.W., Lejuez C., Krueger R.F., Richards J.M., Hill P.L. (2014). What is conscientiousness and how can it be assessed?. Develop. Psych..

[46] DeYoung C.G. (2015). Openness/intellect: A dimension of personality reflecting cognitive exploration. APA Handbook of Personality and Social Psychology, Volume 4: Personality Processes and Individual Differences.

[47] Peiró J.M., Bayona J.A., Caballer A., Di Fabio A. (2020). Importance of work characteristics affects job performance: The mediating role of individual dispositions on the work design-performance relationships. Personal. Individ. Differ..

[48] Di Fabio A. (2017). Positive Healthy Organizations: Promoting well-being, meaningfulness, and sustainability in organizations. Front. Psychol. Org. Psych..

[49] Di Fabio A., Cheung F., Peiró J.-M. (2020). Editorial Special Issue Personality and individual differences and healthy organizations. Personal. Individ. Differ..

[50] Di Fabio A., Giannini M., Loscalzo Y., Palazzeschi L., Bucci O., Guazzini A., Gori A. (2016). The challenge of fostering healthy organizations: An empirical study on the role of workplace relational civility in acceptance of change and well-being. Front. Psychol..

[51] Di Fabio A., Peiró J.M. (2018). Human Capital Sustainability Leadership to promote sustainable development and healthy organizations: A new scale. Sustainability.

[52] Tetrick L.E., Peiró J.M. (2012). Occupational safety and health. The Oxford Handbook of Organizational Psychology.

[53] Gori A., Topino E., Palazzeschi L., Di Fabio A. (2021). Which personality traits can mitigate the impact of the pandemic? Assessment of the relationship between personality traits and traumatic events in the COVID-19 pandemic as mediated by defense mechanisms. PLoS ONE.

[54] Gori A., Topino E., Sette A., Cramer H. (2021). Pathways to post-traumatic growth in cancer patients: Moderated mediation and single mediation analyses with resilience, personality, and coping strategies. J. Affect. Disord..

[55] Gori A., Topino E., Di Fabio A. (2020). The protective role of life satisfaction, coping strategies and defense mechanisms on perceived stress due to COVID-19 emergency: A chained mediation model. PLoS ONE.

[56] Gori A., Arcioni A., Topino E., Craparo G., Lauro Grotto R. (2021). Development of a New Measure for Assessing Mentalizing: The Multidimensional Mentalizing Questionnaire (MMQ). J. Pers. Med..

[57] Haque A., Fernando M., Caputi P. (2020). How is responsible leadership related to the three-component model of organisational commitment?. Int. J. Product. Perform. Manag..

[58] Hetland J., Hetland H., Bakker A.B., Demerouti E. (2018). Daily transformational leadership and employee job crafting: The role of promotion focus. Eur. Manag. J..

[59] Afsar B., Masood M., Umrani W.A. (2019). The role of job crafting and knowledge sharing on the effect of transformational leadership on innovative work behavior. Pers. Rev..

[60] Haque A., Fernando M., Caputi P. (2019). Responsible leadership, affective commitment and intention to quit: An individual level analysis. Leadersh. Organ. Dev. J..

[61] Haque A. (2020). Strategic HRM and organisational performance: Does turnover intention matter?. Int. J. Organ. Anal..

[62] Robertson I., Cooper C.L. (2010). Wellbeing: Productivity and Happiness at Work.

